# Antitumor Effects of Melatonin in Luminal and Triple-Negative Breast Cancer Cells: Metabolic Reprogramming, Redox Regulation, and Cellular Dynamics

**DOI:** 10.3390/cancers18132031

**Published:** 2026-06-23

**Authors:** Roberta Carvalho Cesário, Karolina da Silva Tonon, Vinicius Augusto Simão, Débora Aparecida Pires de Campos Zuccari, Fábio Rodrigues Ferreira Seiva, Maria Luisa Gonçalves Agneis, Russel J. Reiter, Luiz Gustavo de Almeida Chuffa

**Affiliations:** 1 Department of Anatomy, Institute of Biosciences, UNESP—São Paulo State University, Botucatu 18618-689, São Paulo, Brazil; roberta.cesario@unesp.br (R.C.C.); karolina.tonon@unesp.br (K.d.S.T.); vinicius.simao@unesp.br (V.A.S.); 2 Faculdade de Medicina de São José do Rio Preto, São José do Rio Preto 15090-000, São Paulo, Brazil; debora.zuccari@famerp.br; 3 Department of Chemistry and Biochemistry, Institute of Bioscences, UNESP—São Paulo State University, Botucatu 18618-689, São Paulo, Brazil; fabio.seiva@unesp.br (F.R.F.S.); maria.agneis@unesp.br (M.L.G.A.); 4 Department of Cellular and Structural Biology, UT Health San Antonio, San Antonio, TX 78229, USA

**Keywords:** melatonin, breast cancer, cell metabolism, mitochondrial function, redox homeostasis, mitochondrial dysfunction, biogenic amines

## Abstract

**Background/Objectives:** Melatonin is a multifunctional indoleamine with recognized antitumor activity; however, its subtype-specific effects in breast cancer remain incompletely understood. This study aimed to investigate the impact of melatonin on cellular and metabolic processes associated with tumor progression in two human breast cancer cell lines representing distinct molecular subtypes: MCF-7 (luminal A) and MDA-MB-468 (triple-negative). **Methods:** Breast cancer cells were treated with micromolar concentrations of melatonin, and assays were performed to evaluate cell viability, migration, invasion, mitochondrial status, redox balance, protein expression, and biogenic amine profiles. **Results:** Melatonin significantly reduced cell viability, migration, and invasion in both cell lines, with more pronounced effects in MCF-7 cells. At the molecular level, melatonin downregulated key metabolic and hypoxia-related proteins, including GAPDH and HIF-1α, while citrate synthase was selectively reduced in MCF-7 cells, indicating suppression of mitochondrial metabolic capacity. This was accompanied by a reduction in mitochondrial status, reflected by decreased MitoGreen staining. Melatonin also induced redox imbalance, as evidenced by increased lipid peroxidation and protein carbonylation, along with subtype-dependent modulation of antioxidant enzymes. In addition, alterations in biogenic amine profiles were observed, suggesting broader metabolic remodeling. **Conclusions:** Collectively, these findings demonstrate that melatonin exerts subtype-dependent antitumor effects by targeting metabolic, mitochondrial, and redox pathways, supporting further investigation of melatonin as a potential therapeutic adjuvant in breast cancer, while recognizing that the concentrations used in this study exceed physiological circulating levels.

## Simple Summary

Breast cancer is a complex disease that includes different subtypes with distinct behaviors and treatment responses. Identifying compounds that can target tumor growth through multiple mechanisms is essential for improving therapeutic strategies. In this study, we investigated the effects of melatonin, a naturally occurring molecule, on two types of breast cancer cells. We found that melatonin reduced cell growth, migration, and invasion, while also affecting energy production, mitochondrial function, and oxidative balance within the cells. These effects varied depending on the tumor subtype, suggesting that melatonin may act differently according to the biological context of the tumor. Overall, our findings provide experimental evidence that melatonin modulates metabolic and functional pathways in breast cancer cells, supporting further preclinical investigation of its potential therapeutic applications.

## 1. Introduction

Breast cancer is the most common and deadliest malignancy among women worldwide. According to the GLOBOCAN report (2022), the number of new cases is expected to increase substantially in the coming decades [1]. Molecular classification based on the expression of estrogen receptors (ER), progesterone receptors (PR), and human epidermal growth factor receptor 2 (HER2) is essential for therapeutic decision-making and prognosis [2]. Luminal tumors (ER+/PR+), subdivided into subtypes A and B, account for approximately 70% of cases and usually respond to hormone therapy, whereas HER2-positive tumors represent around 20% of cases and may be treated with targeted therapies. Although less frequent (10–20%), triple-negative breast cancer (TNBC; ER−/PR−/HER2−) exhibits aggressive behavior, high metabolic plasticity, and limited therapeutic options [3,4]. Considering the marked biological and metabolic differences between luminal and triple-negative breast cancers, these subtypes were selected as representative models of less aggressive and more aggressive disease phenotypes, respectively. This approach enabled the evaluation of whether melatonin-induced effects are influenced by distinct molecular and metabolic tumor contexts.

Among the hallmarks of cancer, metabolic reprogramming plays a central role in sustaining tumor growth and progression. This process enhances nutrient uptake and energy production while modulating intracellular reactive oxygen species (ROS) levels, thereby supporting deregulated proliferation, immune evasion, tissue invasion, and metastasis [5,6]. Given its fundamental contribution to tumor aggressiveness, metabolic reprogramming represents an attractive target for therapeutic intervention.

Biogenic amines derived from tryptophan metabolism, particularly serotonin and melatonin, are involved in the regulation of cell proliferation, differentiation, and redox balance. These effects are mediated not only by circulating levels of these molecules but also by local synthesis and tissue-specific actions, which have been described in both physiological and pathological conditions [7]. Melatonin (N-acetyl-5-methoxytryptamine) is a lipophilic indoleamine primarily synthesized by the pineal gland, but also produced in mitochondria of perhaps all somatic cells [8]. In healthy cells, melatonin acts as a potent antioxidant by enhancing enzymatic systems responsible for the removal of reactive oxygen and nitrogen species, thereby preventing oxidative damage and cell death [9]. In contrast, in the tumor microenvironment, melatonin may exert antitumor effects, including inhibition of cell proliferation, angiogenesis, and invasion, as well as modulation of immune responses, oxidative stress, and induction of apoptosis [10].

Despite advances in understanding melatonin’s oncostatic properties, its effects on metabolic and mitochondrial pathways across distinct breast cancer subtypes remain insufficiently explored. Comparative analyses focusing on cellular and metabolic processes critical to tumor progression may contribute to the identification of novel therapeutic strategies. Therefore, this study investigated the effects of melatonin on metabolic, mitochondrial, and functional parameters associated with tumor progression in two breast cancer cell lines with distinct molecular profiles, MCF-7 and MDA-MB-468, representing luminal and triple-negative subtypes, respectively. These models were selected because they represent biologically and clinically contrasting breast cancer phenotypes, ranging from a less aggressive hormone-responsive subtype to a highly aggressive triple-negative subtype with marked metabolic plasticity.

Unlike previous studies that have already demonstrated antitumor effects of melatonin on breast cancer cells, the present study expands current knowledge by integrating functional, metabolic, mitochondrial, redox, and biogenic amine analyses within the same experimental framework. In addition, we compared melatonin responses between two biologically distinct breast cancer subtypes and explored the clinical expression patterns of experimentally evaluated metabolic targets using public transcriptomic datasets.

## 2. Materials and Methods

### 2.1. Cell Lines

Two human breast carcinoma cell lines with distinct molecular profiles were used. MCF-7 cell line (ATCC^®^ HTB-22) is derived from metastatic mammary adenocarcinoma and characterized by positivity for estrogen receptor (ER) and progesterone receptor (PR); it is widely used as a representative model of the luminal breast cancer subtype. In contrast, the MDA-MB-468 cell line (ATCC^®^ HTB-132) also originated from metastatic adenocarcinoma, lacks expression of ER, PR, and HER2 receptors, and is representative of the triple-negative breast cancer (TNBC) subtype. The selection of these cell lines was based on their contrasting biological and metabolic characteristics. MCF-7 cells represent a hormone-responsive luminal subtype generally associated with a more favorable prognosis, whereas MDA-MB-468 cells represent an aggressive triple-negative subtype characterized by enhanced metabolic plasticity and limited therapeutic options. This experimental design allowed the investigation of melatonin effects across distinct molecular and metabolic breast cancer contexts. Both cell lines are well-established and frequently utilized in breast cancer research.

### 2.2. Cell Culture and Reagents

Cells were cultured in high-glucose DMEM medium (Gibco, Paisley, UK), supplemented with 10% fetal bovine serum (FBS), 100 U/mL penicillin, and 100 µg/mL streptomycin, at 37 °C in a humidified atmosphere containing 5% CO_2_. The medium was changed every two days, and when cells reached approximately 90% confluency, they were subcultured in 75 cm^2^ flasks (Costar, Corning, NY, USA). For the experiments, cells were detached using a 0.25% trypsin-EDTA solution, centrifuged at 1500 rpm for 5 min, washed with DMEM, and subsequently seeded according to the requirements of each specific assay.

### 2.3. Treatments and Experimental Groups

To investigate the effects of melatonin, the experimental design comprised four groups: (A) MCF-7 control group treated with vehicle; (B) MDA-MB-468 control group treated with vehicle; (C) MCF-7 cells treated with melatonin; and (D) MDA-MB-468 cells treated with melatonin. Melatonin was dissolved in 1% DMSO, which was used as the vehicle control in all experiments. Melatonin concentrations and exposure time were established based on preliminary cell viability assays. Dose–response analyses were conducted using concentrations ranging from 1.25 to 10 µM for MCF-7 cells and from 2 to 10 µM for MDA-MB-468 cells. Based on these assays, 7 µM was defined as the IC_50_ for both cell lines and selected for subsequent experiments.

### 2.4. Cytotoxicity Assay

Cells were seeded in 96-well plates (1 × 10^4^ cells/well) and treated with different concentrations of melatonin for 24 h. Subsequently, MTT solution (5 mg/mL) was added, and plates were incubated for 4 h. The formazan crystals formed were dissolved with DMSO, and absorbance was measured at 550 nm using a microplate reader (Epoch^®^, BioTek, Santa Clara, CA, USA). All experiments were performed in three independent biological replicates, each analyzed in technical triplicate.

### 2.5. Cell Migration Assay

To assess the migratory potential of MCF-7 and MDA-MB-468 cells, the wound healing method, also known as the “scratch assay,” was used. Cells were trypsinized, counted, and seeded at a concentration of 1 × 10^5^ in 6-well plates and incubated at 37 °C for adhesion. Next, a scratch was made on the monolayer using a sterile micropipette tip (1000 µL). The culture medium (DMEM) was removed, and wells were washed with PBS to eliminate non-adherent cells. Melatonin was diluted in complete medium and added to the wells. Cell migration was assessed using images captured with a ZeissAxiovert^®^ microscope at 0, 6, 12, and 24 h. The wound area was measured using ImageJ software (version 1.45; National Institutes of Health, Bethesda, MD, USA). Wound closure was calculated relative to the initial wound area (0 h) according to the following equation: Wound Closure (%) = [(A_0_ − At)/A_0_] × 100, where A_0_ represents the wound area at 0 h and At represents the wound area at the corresponding experimental time point. Quantification was performed using ImageJ software, based on the percentage of closed area. A positive control was maintained without melatonin exposure, while the negative control was characterized by the absence of fetal bovine serum (FBS). The assay was performed in three independent biological experiments.

### 2.6. Cell Invasion Assay

Cell invasion was assessed using 24-well plates equipped with Transwell chambers. Each insert was coated with 56 µL of Geltrex^®^, forming a thin layer over the bottom polyethylene terephthalate (PET) membrane with 8 µm pores. MCF-7 and MDA-MB-468 cells (6 × 10^4^ cells per well) were seeded into the upper chamber in serum-free medium to minimize proliferation and stimulate migration. The lower chamber contained medium supplemented with 5% FBS to serve as a chemoattractant. Plates were incubated at 37 °C in a humidified CO_2_ atmosphere for 24 h. After incubation, non-invading cells on the upper side of the membrane were gently removed by scraping, while the cells that invaded the membrane were fixed in 10% methanol for 10 min. Invasive cells were visualized using an inverted microscope ZeissAxiovert^®^, Carl Zeiss, Oberkochen, Germany) and quantified in ImageJ by counting the number of cells that had crossed the membrane. Cell invasion analyses were performed in three independent biological experiments.

### 2.7. Protein Analysis and Quantification by Western Blot

Following melatonin treatment, MCF-7 and MDA-MB-468 cells were seeded at a density of 5 × 10^5^ cells per well. After an appropriate incubation period, the culture medium was removed, and the wells were washed with cold PBS to eliminate non-adherent cells. RIPA lysis buffer with a protease inhibitor cocktail (Sigma CO, Saint Louis, MO, USA) was added to the wells, and the plates were stored at −20 °C. After a minimum of 24 h and up to 7 days, the wells were scraped, and the lysates were collected. The samples were centrifuged to remove debris, and the supernatants were used for protein quantification using the Bradford assay. Equal amounts of protein (40 μg per sample) were solubilized and loaded onto a 4–20% polyacrylamide gel (SDS-PAGE). After electrophoresis, proteins were electrotransferred (300 mA) onto a nitrocellulose membrane (BioRad, Hercules, CA, USA). The membrane was blocked with 3% (*w*/*v*) BSA and incubated overnight with the following monoclonal primary antibodies diluted in 1% BSA: anti-hypoxia-inducible factor 1-alpha (HIF-1α, 1:500), anti-glyceraldehyde-3-phosphate dehydrogenase (GAPDH, 1:500), anti-citrate synthase (CS, 1:500), and anti-α-tubulin (1:500) (ABCAM, Cambridge, UK). The membrane was washed 3 times in basal solution (TBS-Tween 1% [*v*/*v*]) and incubated with the secondary antibody for 1 h. After three additional washes with basal solution, the signal was developed using the ECL^®^ Selected Western Blotting Detection Reagent (GE Healthcare, Uppsala, Sweden). Image acquisition was performed with the G-Box transilluminator system, and densitometric quantification of the bands was carried out using ImageJ software. Band intensity was quantified based on the integrated optical density index (IOD) and normalized to α-tubulin as the loading control. Western blot analyses were performed using protein extracts obtained from three independent biological experiments.

### 2.8. Analysis of Oxidative Stress Markers and Antioxidant Enzyme Activities

Oxidative stress in melatonin-treated cells was assessed by measuring specific biomarkers and antioxidant enzyme activities. Malondialdehyde (MDA), protein carbonyls, and reduced glutathione (GSH) levels were quantified by spectrophotometry at 532 nm, 370 nm, and 412 nm, respectively. Antioxidant enzyme activities were measured as follows: catalase (CAT) activity was determined by monitoring the decomposition of H_2_O_2_ at 240 nm; superoxide dismutase (SOD) activity by the inhibition of nitroblue tetrazolium (NBT) reduction at 560 nm; glutathione peroxidase (GSH-Px) activity by the reduction in H_2_O_2_ at 412 nm; and glutathione S-transferase (GSH-ST) activity by the conjugation of 1-chloro-2,4-dinitrobenzene (CDNB) at 340 nm. All assays were performed in three independent biological experiments, each analyzed in technical triplicate.

### 2.9. Enzymatic Activities of LDH, G6PDH, and Citrate Synthase

The activities of lactate dehydrogenase (LDH), glucose-6-phosphate dehydrogenase (G6PDH), and citrate synthase (CS) were determined using colorimetric enzymatic assays with commercial kits, according to the manufacturer’s instructions. Analyses were performed using cellular extracts obtained from MCF-7 and MDA-MB-468 cells under control conditions and after melatonin treatment.

LDH and G6PDH activities were quantified based on NADH formation, monitored by absorbance at 450 nm. Citrate synthase activity was assessed through the formation of 5-thio-2-nitrobenzoate (TNB), with absorbance measured at 412 nm. For all assays, absorbance readings were acquired in microplates at regular time intervals, allowing calculation of the change in absorbance (ΔAbs) between initial and final time points within the linear phase of the reaction.

Standard curves were constructed for each assay using known concentrations of NADH (for LDH and G6PDH) or reduced glutathione (GSH) (for citrate synthase), enabling conversion of ΔAbs values into product formation (nmol). Enzymatic activity was subsequently calculated and expressed as mU/mL, taking into account reaction time and sample volume per well. All samples were analyzed in duplicate.

### 2.10. Assessment of Mitochondrial Fluorescence (MitoGreen)

Mitochondrial fluorescence was assessed by staining with a mitochondria-specific fluorescent dye (MitoGreen), using a commercial kit, according to the manufacturer’s instructions. Analyses were performed in the MCF-7 and MDA-MB-468 cells under control conditions and after melatonin treatment.

Cells were incubated with the dye for 60 min at 37 °C, protected from light. After incubation, fluorescence images were acquired in the green channel and qualitatively analyzed based on the relative fluorescence intensity and the distribution pattern of mitochondrial staining among the experimental groups. Fluorescence analyses were performed in three independent biological experiments.

### 2.11. Polyamine Extraction

Polyamine and biogenic amine analyses were performed using samples obtained from three independent biological experiments. Polyamines were extracted from 200 mg of dried sample by adding 3 mL of perchloric acid (5% HClO_4_) followed by thorough homogenization. The mixture was subjected to an ice-cold ultrasonic bath for 30 min and then centrifuged at 3200 rpm for 10 min. The resulting supernatant was collected and stored under refrigeration. For derivatization, 200 μL of the supernatant was mixed with carbonate buffer and dansyl chloride, then incubated at 60 °C for 1 h. Subsequently, proline was added, and the reaction mixture was maintained in the dark for an additional 1 h. The solution was extracted with toluene, vigorously shaken, centrifuged, and the organic phase was transferred to a plastic tube, evaporated under a nitrogen stream, and resuspended in acetonitrile. After a second centrifugation step, the supernatant was filtered and transferred to vials for high-performance liquid chromatography (HPLC) analysis.

### 2.12. Identification of Polyamines and Amines by HPLC

The identification and quantification of polyamines and amines in the extracted samples were performed using HPLC on a Thermo Scientific Ultimate 3000 system equipped with a UV-visible detector, Thermo Fisher Scientific, Waltham, MA, USA. Prior to injection, cell extracts were pre-filtered using 0.45 μm membranes to remove particulates. Separation was carried out on a C18 reversed-phase column, with detection set at 254 nm, or adjusted according to the specific absorbance characteristics of each amine. Elution conditions were optimized using a methanol-based mobile phase optimized to achieve efficient separation of the analytes. Identification of compounds was based on retention times compared with commercial standards, and quantification was determined by interpolation from standard calibration curves.

### 2.13. Diagnostic and Clinical Relevance Analysis of Melatonin-Modulated Genes

To ensure consistency between the experimental and clinical components of the study, only genes directly evaluated in vitro were included in the transcriptomic analyses. Thus, the in silico investigation was designed to explore the clinical expression patterns of experimentally assessed targets rather than to perform a broad exploratory analysis of metabolic genes.

We reanalyzed publicly available transcriptomic data to explore the expression of melatonin-modulated metabolic genes in clinical breast cancer datasets. The expression patterns of melatonin-modulated metabolic genes (HIF1A, LDHA, G6PD, and CS) were evaluated using transcriptomic data from breast cancer clinical cohorts. These genes were selected because they represent complementary metabolic pathways involved in tumor progression, including hypoxia signaling (HIF1A), glycolysis (LDHA), pentose phosphate pathway activity and redox regulation (G6PD), and mitochondrial oxidative metabolism (CS). The association between gene expression and tumor progression was first evaluated using public RNA-seq data from the TNMplot platform (https://tnmplot.com/, accessed on 2 January 2025), comprising normal breast tissue, primary tumors, and metastatic lesions. Gene expression levels were compared across normal, primary tumor, and metastatic samples, and results were expressed as fold change values.

The discriminative performance of these genes was subsequently evaluated in an independent TCGA-BRCA cohort using RNA-seq data retrieved via the TCGAbiolinks package in R. Normalized FPKM values were log2-transformed, and samples from normal breast tissue and primary tumors were included. A metabolic gene signature was generated based on the mean expression of HIF1A, LDHA, G6PD, and CS for each sample.

The ability of the gene signature to discriminate tumor from normal samples was evaluated by receiver operating characteristic (ROC) curve analysis using the pROC package. Diagnostic performance was quantified by the area under the curve (AUC), with sensitivity and specificity determined using the Youden index. Subtype-specific analyses were performed according to PAM50 classification, focusing on Luminal A and triple-negative breast cancer subtypes.

### 2.14. Statistical Analysis

Data were analyzed by One-way analysis of variance (ANOVA), followed by Tukey’s multiple comparison test for parametric distributions. For non-parametric data, the Kruskal–Wallis test was applied, followed by Dunn’s post hoc test. Results were expressed as the mean ± SEM and presented in graphs. A significance level of 5% (*p* < 0.05) was considered. Unless otherwise stated, all experiments were performed in three independent biological replicates, with technical replicates used when appropriate for each assay. Statistical analyses were performed using GraphPad Prism software (version 9, GraphPad Software, San Diego, CA, USA).

## 3. Results

### 3.1. Melatonin Treatment Reduced Viability, Migration, and Invasion in MCF-7 and MDA-MB-468 Breast Carcinoma Cells

**Figure 1:**
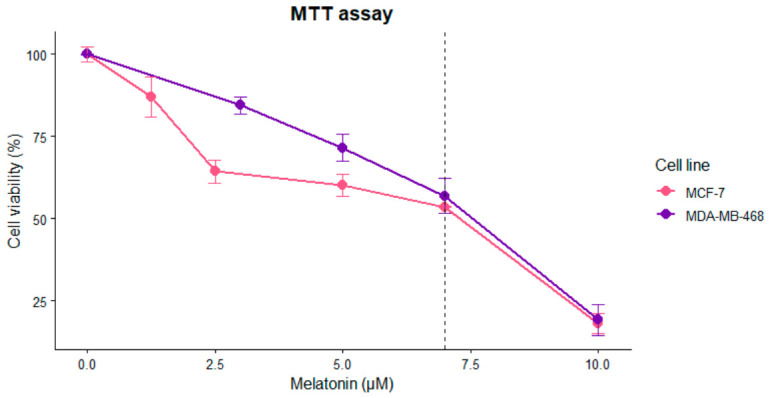
Cell viability of MCF-7 and MDA-MB-468 cells after 24 h of treatment with increasing concentrations of melatonin, assessed by the MTT assay. Data are expressed as mean ± SEM from three independent experiments. The dashed vertical line indicates the IC_50_ (7 µM). Statistical analysis was performed using one-way ANOVA followed by Tukey’s post hoc test, with *p* < 0.05 compared with the respective control.

Cell viability was assessed using the MTT assay in technical and biological triplicate. After 24 h of treatment with increasing concentrations of melatonin (1.25–10 µM), a reduction in cell viability was observed in the MCF-7 and MDA-MB-468 cell lines (Figure 1). Based on the dose–response profile, the 50% inhibitory concentration (IC_50_) was estimated at approximately 7 µM for both cell lines, and this concentration was selected for subsequent experiments.

**Figure 2:**
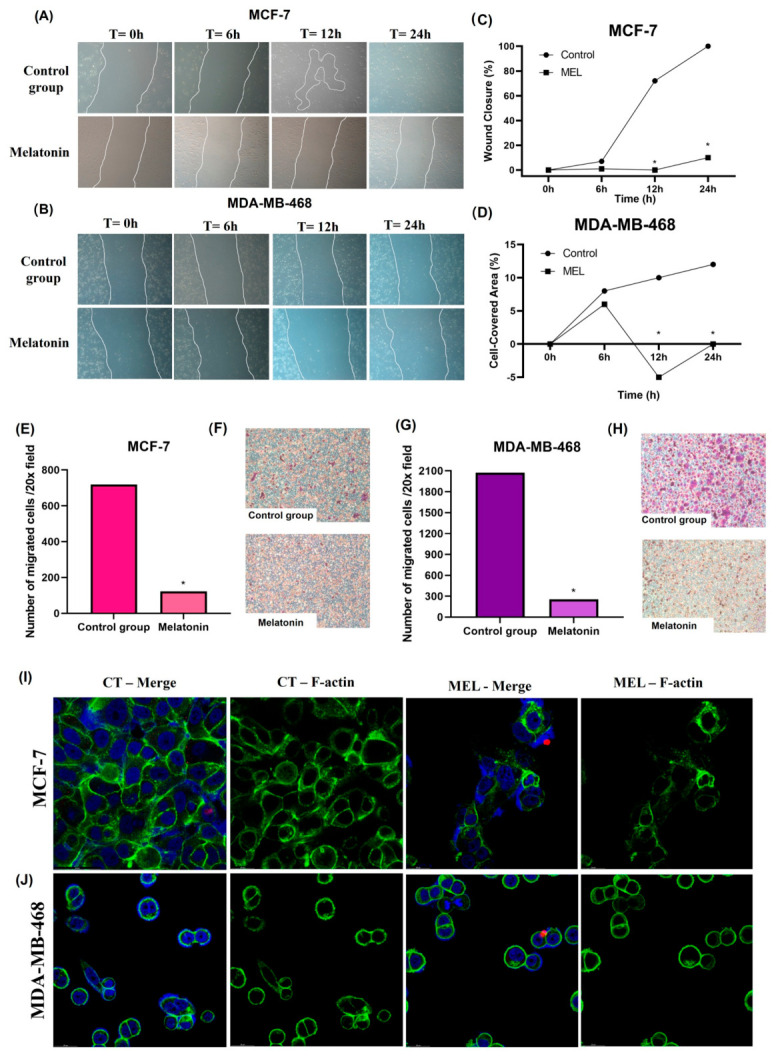
Effect of melatonin on the migratory and invasive capacities of MCF-7 and MDA-MB-468 breast cancer cells. (**A**,**B**) Representative images of the wound healing assay in MCF-7 (**A**) and MDA-MB-468 (**B**) cells at different time points after scratch formation, in the presence or absence of melatonin. (**C**,**D**) Quantitative analysis of wound closure at 0, 6, 12, and 24 h post-scratch for MCF-7 (**C**) and MDA-MB-468 (**D**) cells. (**E**–**H**) Effects of melatonin on cell invasion assessed by transwell assay. (**E**) Quantification of invasive MCF-7 cells and (**F**) representative images of invaded cells after treatment. (**G**) Quantification of invasive MDA-MB-468 cells and (**H**) representative images of invaded cells after treatment. Phalloidin (green) and DAPI (blue) staining in MCF-7 (**I**) and MDA-MB-468 (**J**). Pink bars represent MCF-7 cells, whereas purple bars represent MDA-MB-468 cells. Darker shades indicate control groups and lighter shades indicate melatonin-treated groups. Data are expressed as mean ± SEM or percentage, as indicated. * *p* < 0.05 compared to the respective control group. Statistical significance was determined using Student’s *t*-test.

To investigate the impact of melatonin treatment on the migratory potential of MCF-7 and MDA-MB-468 breast cancer cells, a wound healing assay was performed. Cells were seeded in 6-well culture plates, and immediately following treatment exposure, wound closure was monitored by capturing images at 0, 6, 12, and 24 h. Notably, in MCF-7 cells, the control group exhibited complete wound closure within 24 h, whereas the melatonin-treated group showed only 10% closure during the same period (Figure 2A). A significant reduction in migration was already evident at 12 h in the treated group, compared to the control (Figure 2A,C). In MDA-MB-468 cells, the control group achieved 12% closure after 24 h, while the melatonin-treated group initially showed 8% closure within the first 6 h. However, this effect was not sustained, and at the end of 24 h, wound closure in the treated group had decreased to 0% (Figure 2B,D). These findings indicate that melatonin impairs the migratory capacity of breast cancer cells. In MDA-MB-468 cells, the reduction in wound closure observed after treatment may be associated with loss of cell adhesion and/or decreased cell viability, as supported by the cytotoxic effects and morphological alterations observed following melatonin exposure.

The invasive potential of MCF-7 and MDA-MB-468 breast cancer cells was assessed with the aid of a cell invasion assay using Matrigel-coated transwell inserts in 24-well plates. Following 24 h of treatment with melatonin, the number of cells that crossed the membrane was quantified. A significant reduction in invasive potential was observed in both cell lines treated with melatonin compared to their respective controls, with a reduction of 82.9% in MCF-7 cells (Figure 2E,F) and 87.6% in MDA-MB-468 cells (Figure 2G,H). These findings indicate that melatonin effectively suppresses the invasive behavior of these breast cancer cells.

To further investigate the mechanisms underlying the reduced migratory and invasive capacities, actin cytoskeleton organization was evaluated by phalloidin staining. In MCF-7 cells (Figure 2I), melatonin treatment led to reduced cell spreading, increased cell rounding, and redistribution of actin filaments toward the cell periphery. In MDA-MB-468 cells (Figure 2J), a more pronounced disruption was observed, with decreased actin organization, altered morphology, and increased cellular isolation.

### 3.2. Melatonin Reduces Protein Levels of GAPDH, HIF-1α, and CS Associated with Tumor Progression

**Figure 3:**
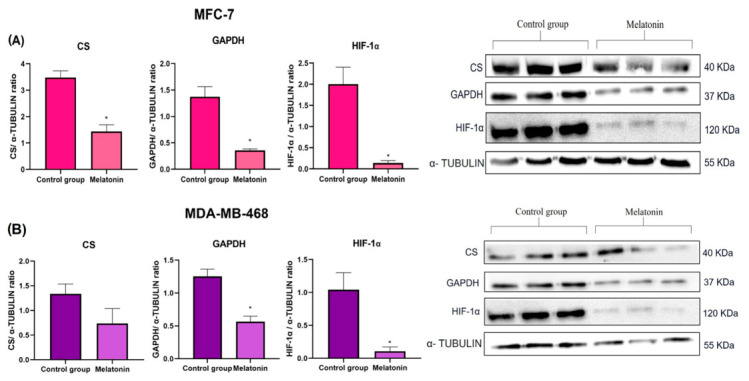
Melatonin’s effect on the expression of citrate synthase (CS), glyceraldehyde-3-phosphate dehydrogenase (GAPDH), and hypoxia-inducible factor 1-alpha (HIF-1α) in MCF-7 and MDA-MB-468 breast cancer cells. Representative Western blots and corresponding densitometric analyses for CS, GAPDH, and HIF-1α normalized to α-tubulin in MCF-7 (**A**) and MDA-MB-468 (**B**) cells after melatonin treatment. Pink bars represent MCF-7 cells, whereas purple bars represent MDA-MB-468 cells. Darker shades indicate control groups and lighter shades indicate melatonin-treated groups. Data are presented as mean ± SEM. * *p* < 0.05 compared to the respective control group (Student’s *t*-test). Uncropped images are provided in the Supplementary Document S1.

To evaluate the potential impact of melatonin on proteins involved in tumor-promoting processes, the expression levels of HIF-1α, GAPDH, and CS were analyzed by Western blot. Densitometric analysis revealed a marked reduction in HIF-1α and GAPDH expression in melatonin-treated cells compared to controls in both MCF-7 and MDA-MB-468 cells (Figure 3), indicating a potential role for melatonin in suppressing key metabolic and hypoxia-related pathways in breast cancer.

Regarding CS enzyme levels, melatonin treatment significantly reduced its expression in the MCF-7 cell line (Figure 3A). In contrast, no statistically significant difference was observed in the MDA-MB-468 cells (Figure 3B). Collectively, these findings suggest that melatonin modulates key proteins involved in tumor progression, reinforcing its potential role as a regulatory agent of tumor metabolism in breast cancer.

### 3.3. Melatonin Differentially Modulates Metabolic Enzymes and Mitochondrial Status in Breast Cancer Cells

**Figure 4:**
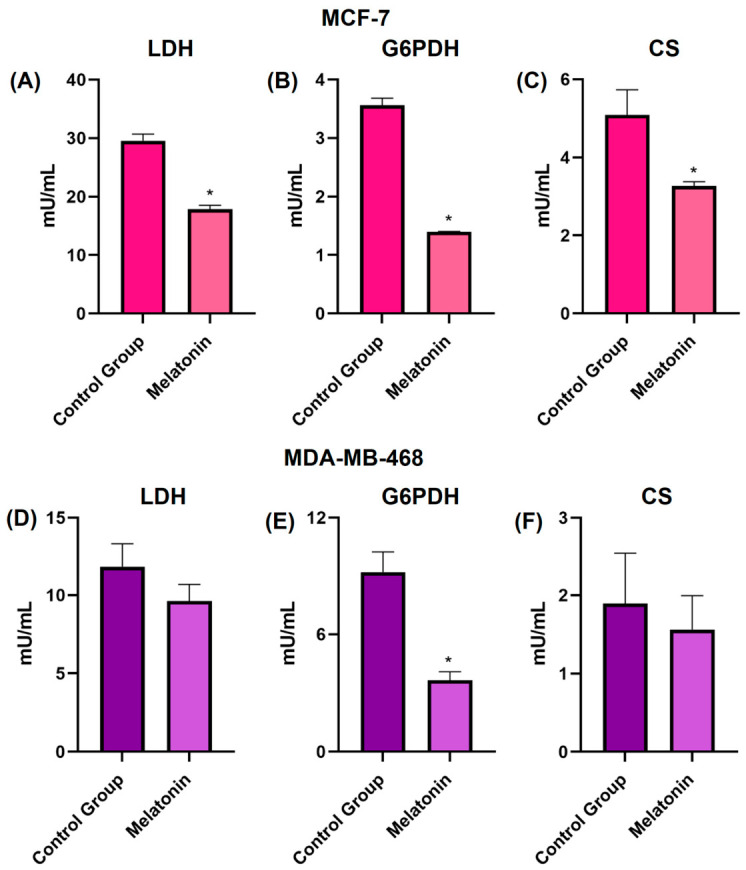
Enzymatic activity of lactate dehydrogenase (LDH), glucose-6-phosphate dehydrogenase (G6PDH), and citrate synthase (CS) in MCF-7 and MDA-MB-468 breast cancer cell lines under control conditions (Control Group) and after melatonin treatment (Melatonin). (**A**–**C**) LDH (**A**), G6PDH (**B**), and CS (**C**) activities in the MCF-7 cell line. (**D**–**F**) LDH (**D**), G6PDH (**E**), and CS (**F**) activities in the MDA-MB-468 cell line. Data are presented as mean ± SEM from two independent replicates. Comparisons between control and melatonin-treated groups were performed using an unpaired Student’s *t*-test. * *p* < 0.05 versus the control group. Pink bars represent MCF-7 cells, whereas purple bars represent MDA-MB-468 cells. Darker shades indicate control groups and lighter shades indicate melatonin-treated groups.

The activity of the enzymes LDH, G6PDH, and CS was evaluated in MCF-7 and MDA-MB-468 cell lines under control conditions and after melatonin treatment (Figure 4). In MCF-7 cells, melatonin treatment resulted in a significant reduction in LDH activity compared to the control group (Figure 4A). Consistently, a significant decrease in G6PDH activity was also observed (Figure 4B). In addition, CS activity was significantly reduced in melatonin-treated cells (Figure C). In the MDA-MB-468 cell line, LDH activity showed a modest reduction following melatonin treatment; however, this change did not reach statistical significance (Figure 4D). In contrast, G6PDH activity was significantly reduced in treated cells (Figure 4E), whereas CS activity did not differ significantly between control and melatonin-treated groups (Figure 4F).

**Figure 5:**
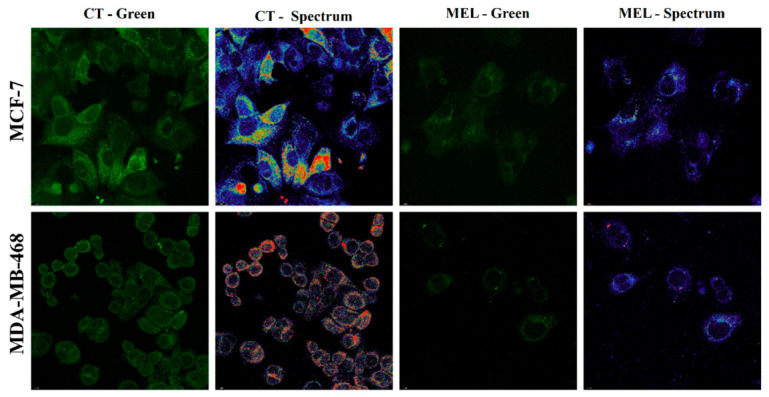
Mitochondrial fluorescence assessed by MitoGreen staining in MCF-7 and MDA-MB-468 cell lines under control (CT) and melatonin-treated (MEL) conditions. CT-Green and MEL-Green confocal images correspond to the original MitoGreen signal in the green channel, whereas CT-Spectrum and MEL-Spectrum images represent the pseudocolor (spectrum) display of fluorescence intensity, in which warm colors indicate higher signal intensity and cool colors indicate lower signal intensity.

To further investigate whether these metabolic alterations were accompanied by changes in mitochondrial status, mitochondrial fluorescence was assessed using MitoGreen staining in both cell lines under control (CT) and melatonin-treated (MEL) conditions (Figure 5). Images were analyzed in the green channel and additionally displayed using pseudo-color (spectrum) representation to facilitate visualization of variations in fluorescence intensity.

Under control conditions, both cell lines exhibited intense and widely distributed mitochondrial fluorescence, with these organelles being distributed throughout the cytoplasm. MCF-7 cells displayed a more diffuse staining pattern, whereas MDA-MB-468 cells showed a more punctate and heterogeneous distribution. Following melatonin treatment, a reduction in mitochondrial fluorescence intensity was observed in both cell lines, evident in both the green channel and the pseudo-color representation, with a predominance of cooler tones in the spectrum images. Additionally, a decrease in highly fluorescent areas was noted, with this effect being more pronounced in the MDA-MB-468 cell line.

### 3.4. Melatonin Modulates Antioxidant Enzymes and Oxidative Stress Responses

**Figure 6:**
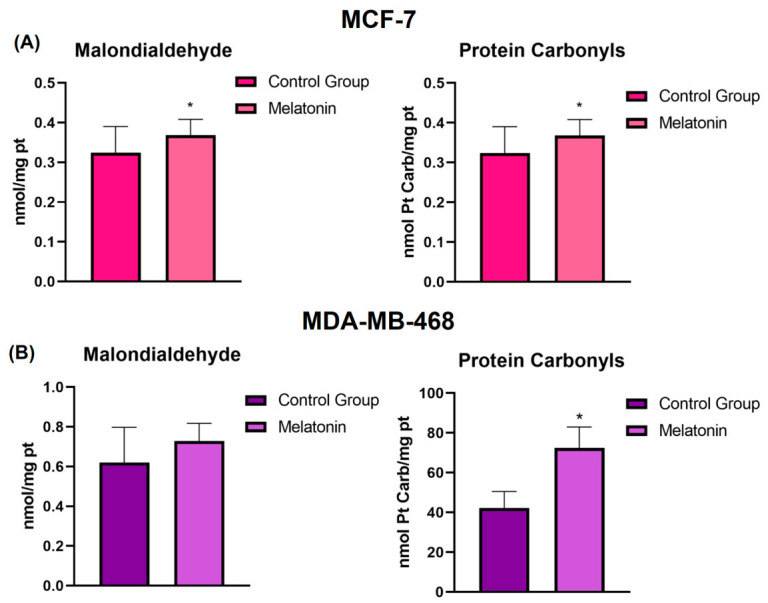
Melatonin’s actions on oxidative stress markers in breast cancer cells. Comparison of malondialdehyde (MDA) and carbonylated protein levels between control and melatonin-treated MCF-7 cells (**A**) and MDA-MB-468 cells (**B**). Data are expressed as mean ± SEM. * *p* < 0.05 vs. control group. Student’s *t* test. Pink bars represent MCF-7 cells, whereas purple bars represent MDA-MB-468 cells. Darker shades indicate control groups and lighter shades indicate melatonin-treated groups.

Melatonin treatment led to a significant increase in malondialdehyde (MDA, previously identified) and carbonylated protein levels in the MCF-7 cell line (*p* < 0.05), indicating enhanced oxidative stress and associated cellular damage (Figure 6A). In MDA-MB-468 cells, although MDA levels remained unchanged, a significant rise in the concentration of carbonylated proteins was observed (*p* < 0.05), suggesting that melatonin also induces oxidative protein damage in this more aggressive subtype, albeit through a distinct response profile. These findings reinforce melatonin’s potential to modulate the intracellular redox balance, which may contribute to its antitumor activity in breast cancer cells.

**Figure 7:**
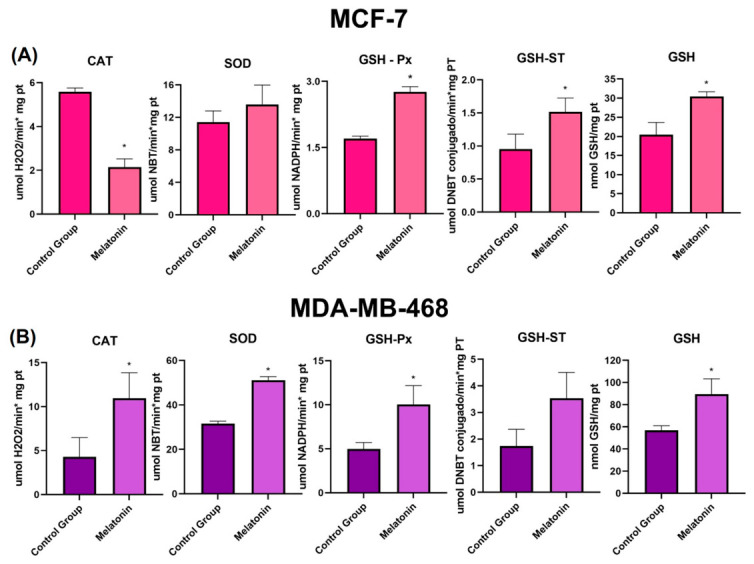
Melatonin’s effects on antioxidant enzyme activity in breast cancer cells. Enzymatic activities of CAT, SOD, GSH-Px, GSH-ST, and GSH in control and melatonin-treated MCF-7 cells (**A**) and MDA-MB-468 cells (**B**). Data are expressed as mean ± SEM. * *p* < 0.05 vs. control group. Student’s *t* test. CAT: Catalase, SOD: Superoxide Dismutase, GSH-Px: Glutathione Peroxidase, GSH-ST: Glutathione S-Transferase, and GSH: Reduced Glutathione. Pink bars represent MCF-7 cells, whereas purple bars represent MDA-MB-468 cells. Darker shades indicate control groups and lighter shades indicate melatonin-treated groups.

The levels of CAT, SOD, GSH-Px, GSH-ST, and GSH were analyzed to assess melatonin-induced modulation of the antioxidant system. In the MCF-7 cell line, a reduction in CAT levels was observed, accompanied by significant increases in GSH-ST and GSH-Px activities, as well as elevated GSH levels (Figure 7A). In the MDA-MB-468 cell line, all parameters showed significant increases, except for GSH-ST, which remained unchanged, indicating a distinct response of the triple-negative subtype to melatonin treatment (Figure 7B).

### 3.5. Melatonin Alters the Biogenic Amine Profile in MCF-7 and MDA-MB-468 Cells

**Figure 8:**
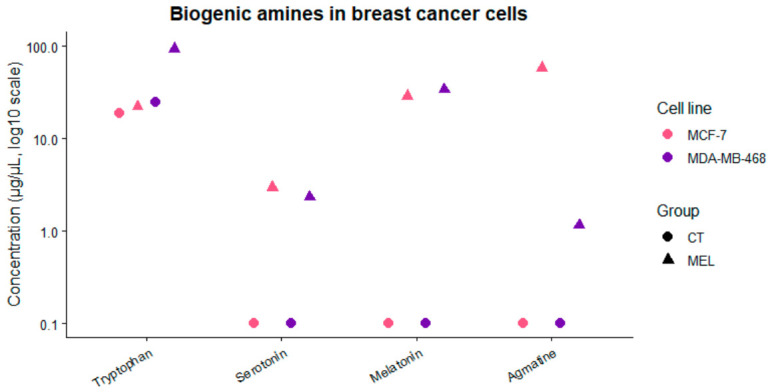
Biogenic amines in breast cancer cells. Concentrations of selected biogenic amines in MCF-7 and MDA-MB-468 cells under control (CT) and melatonin-treated (MEL) conditions. Data are shown on a log10 scale. Non-detected (ND) values were plotted using a pseudocount (0.1 µg/µL) to allow visualization on the logarithmic scale.

Following melatonin treatment, biogenic amines and polyamines were extracted and analyzed by HPLC in MCF-7 and MDA-MB-468 breast cancer cell lines (Figure 8). Tryptophan was detected in both control and melatonin-treated groups in both cell lines; however, a statistically significant increase in tryptophan levels was observed after melatonin treatment specifically in MDA-MB-468 cells. In contrast, agmatine, melatonin, and serotonin were not detected in control groups of either cell line, but became detectable after melatonin treatment, reaching biologically relevant concentrations. These results suggest that melatonin is taken up by cells and modulates the intracellular amine profile, potentially influencing metabolic and signaling pathways in breast cancer cells.

### 3.6. Expression Patterns and Discriminative Performance of Melatonin-Modulated Metabolic Genes

**Figure 9:**
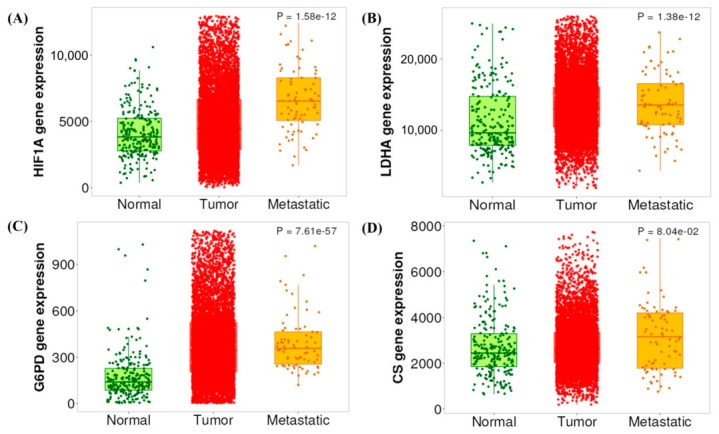
Expression profiles of metabolic genes modulated by melatonin across breast cancer progression stages. Boxplots show the expression levels of (**A**) HIF1A, (**B**) LDHA, (**C**) G6PD, and (**D**) CS in normal breast tissue, primary tumors, and metastatic lesions based on RNA-seq data retrieved from the TNMplot platform. Green boxplots represent normal breast tissue samples, red dots represent primary tumor samples, and orange boxplots represent metastatic lesions. Individual dots correspond to individual samples. Statistical significance between groups is indicated by the corresponding *p*-values.

Based on TCGA data, the expression of HIF1A, LDHA, G6PD, and CS differed among normal breast tissue, primary tumors, and metastatic lesions (Figure 9). HIF1A, LDHA, and G6PD exhibited significantly higher expression in primary tumors compared with normal tissue, with maintenance or further increases in metastatic samples. In contrast, CS expression did not differ significantly between normal breast tissue and primary tumors, but was markedly increased in metastatic lesions, indicating a distinct expression pattern across disease stages.

Comparison between primary tumors and metastatic lesions revealed the persistence of tumor-associated expression patterns for HIF1A, LDHA, and G6PD, whereas CS showed a metastasis-associated increase, suggesting differential regulation of this gene during disease progression.

**Figure 10:**
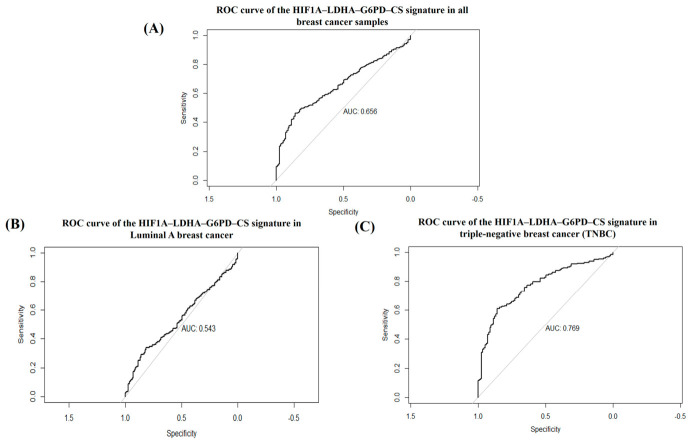
Diagnostic performance of the HIF1A–LDHA–G6PD–CS metabolic gene signature in breast cancer. Receiver operating characteristic (ROC) curves show the ability of the gene signature to discriminate tumor from normal samples in (**A**) the overall breast cancer cohort, (**B**) the Luminal A subtype, and (**C**) the triple-negative breast cancer (TNBC) subtype. The area under the curve (AUC) is indicated in each panel.

Evaluation of the discriminative performance of the gene signature comprising HIF1A, LDHA, G6PD, and CS revealed a moderate ability to distinguish tumor samples from normal breast tissue in the overall breast cancer cohort, as indicated by receiver operating characteristic (ROC) curve analysis (Figure 10A). These findings suggest that the selected metabolic genes display differential expression patterns between normal and tumor tissues, consistent with metabolic alterations commonly observed in breast cancer.

Subtype-stratified analyses revealed distinct discriminative patterns. In the Luminal A subtype, the signature exhibited limited ability to distinguish tumor from normal samples (Figure 10B), whereas in the triple-negative subtype, a greater discriminative capacity was observed, as indicated by a higher AUC value (Figure 10C).

It is important to note that this analysis was intended to explore transcriptomic expression patterns of melatonin-modulated metabolic genes in clinical datasets and should not be interpreted as evidence of biological causality or therapeutic relevance.

## 4. Discussion

This study investigated the effects of melatonin on human breast carcinoma cells representing distinct molecular subtypes, MCF-7 and MDA-MB-468. Breast cancer remains the most common and deadliest malignancy among women worldwide [1], and tumor progression is strongly supported by metabolic adaptations that sustain proliferation, invasion, and survival [11]. In this context, elucidating how melatonin interferes with tumor metabolism and associated cellular processes may contribute to the development of complementary therapeutic strategies. Consistent with previous reports, melatonin reduced cell viability, migration, and invasion in both breast cancer cell lines, confirming antitumor effects that have already been documented in the literature. Although several studies have reported antitumor effects of melatonin in breast cancer models, the novelty of the present study lies in the integration of functional, metabolic, mitochondrial, redox, and biogenic amine analyses under a unified experimental setting. In addition, the study directly compared luminal and triple-negative breast cancer cells, allowing the identification of subtype-dependent responses to melatonin, and integrated experimentally evaluated metabolic targets with clinical transcriptomic datasets. Furthermore, the comparative evaluation of luminal and triple-negative breast cancer cells revealed subtype-dependent responses that may have been overlooked in studies focused on a single tumor subtype.

Cell viability assays demonstrated that micromolar concentrations of melatonin (2.5–10 µM) significantly reduced the viability of both cell lines, with 7 µM selected for subsequent analyses. While several studies report cytotoxic effects of melatonin at millimolar concentrations [12,13], the present findings indicate that lower, biologically relevant doses are sufficient to impair tumor cell viability and aggressiveness. This observation is consistent with previous reports showing that micromolar melatonin modulates oncogenic signaling pathways and angiogenic mediators in breast cancer cells, including triple-negative subtypes [14], reinforcing its role as an active metabolic modulator rather than a nonspecific cytotoxic agent. In addition, earlier studies have demonstrated that even nanomolar concentrations of melatonin (1 nM) exert oncostatic effects in estrogen receptor–positive breast cancer cells, which are critically dependent on intracellular glutathione availability. Pharmacological inhibition of glutathione synthesis abolishes the antiproliferative effects of melatonin, while exogenous glutathione restores this response, highlighting the importance of redox balance in mediating melatonin activity. These findings further support the notion that melatonin’s antitumor effects are tightly linked to cellular metabolic and redox regulation, rather than direct cytotoxicity [15].

Beyond effects on viability, melatonin significantly reduced the migratory and invasive capacities of both MCF-7 and MDA-MB-468 cells. Migration and invasion are critical events in tumor progression and metastatic dissemination, involving cytoskeletal remodeling, extracellular matrix degradation, and epithelial–mesenchymal transition. The inhibitory effects observed here are consistent with previous studies demonstrating that melatonin interferes with motility-associated pathways and downregulates matrix metalloproteinases across different breast cancer subtypes [13,16], suggesting that melatonin may contribute to the modulation of processes associated with aggressive tumor behavior.

At the molecular level, melatonin promoted a coordinated suppression of metabolic and mitochondrial markers. Reduced expression of GAPDH, CS, and HIF-1α was observed following treatment, indicating impairment of glycolytic flux, mitochondrial bioenergetic capacity, and hypoxia-associated signaling. GAPDH overexpression has been linked to poor prognosis and enhanced proliferation in breast cancer [17], while CS plays a central role in sustaining mitochondrial ATP production and biosynthetic demands [18]. The reduction in CS expression and activity, together with altered mitochondrial fluorescence patterns, supports a model of mitochondrial metabolic repression rather than classical metabolic reprogramming, in line with previous reports describing melatonin-induced mitochondrial dysfunction in cancer cells [19,20].

Consistent with these findings, the activity of key metabolic enzymes, including LDH, G6PDH, and CS, was differentially modulated by melatonin, with more pronounced effects in MCF-7 cells compared to MDA-MB-468 cells. These subtype-dependent responses likely reflect intrinsic differences in metabolic flexibility and redox handling between luminal and triple-negative breast cancer cells, reinforcing the importance of tumor context in determining sensitivity to metabolic interventions.

Melatonin also affected redox homeostasis, inducing oxidative stress in tumor cells as evidenced by increased lipid peroxidation and protein carbonylation, particularly in MCF-7 cells. Although melatonin is widely recognized as a potent antioxidant in normal tissues, its pro-oxidant effects in cancer cells have been associated with selective antitumor activity [21]. This apparent paradox is supported by studies demonstrating a dual role of melatonin in regulating intracellular redox balance, in which antiproliferative effects are associated with reduced reactive oxygen species (ROS) levels and enhanced antioxidant defenses, whereas cytotoxic effects are linked to increased ROS production and depletion of antioxidant systems, including glutathione [22]. The differential modulation of antioxidant enzymes between the two cell lines suggests adaptive, subtype-specific responses to redox imbalance, consistent with previous studies employing low micromolar concentrations of melatonin [23]. In this context, tumor cell fate appears to depend on the redox environment induced by melatonin, shifting between antioxidant and pro-oxidant states according to cellular context and metabolic profile.

In addition to energy metabolism, melatonin modulated biogenic amines and related metabolic intermediates, indicating broader metabolic remodeling. Alterations in histamine- and tryptophan-related pathways suggest potential interactions with signaling and survival mechanisms previously associated with tumor progression and patient outcomes [24,25]. Together, these data highlight the multifaceted metabolic impact of melatonin beyond classical bioenergetic pathways.

Overall, melatonin exerts pleiotropic effects on breast cancer cells, impairing proliferation, migration, invasion, metabolism, mitochondrial function, and redox balance. The distinct responses observed between MCF-7 and MDA-MB-468 cells underscore the influence of molecular subtype and metabolic context on melatonin sensitivity, supporting its potential as a therapeutic adjuvant.

Importantly, the clinical relevance of these experimental findings was further supported by complementary in silico analyses using breast cancer patient cohorts. Metabolic genes associated with hypoxia, glycolysis, and redox control, including HIF1A, LDHA, and G6PD, are frequently upregulated in breast tumors and remain altered during disease progression, particularly in aggressive molecular subtypes [26,27]. In contrast, CS expression does not differ significantly between normal breast tissue and primary tumors but is selectively increased in metastatic lesions, suggesting a role in advanced stages of tumor progression, as reported for other cancer types [28]. In this context, melatonin-induced suppression of CS supports a model of mitochondrial metabolic repression, potentially limiting the bioenergetic capacity required for tumor progression and dissemination [19]. Furthermore, analysis of a metabolic gene signature combining HIF1A, LDHA, G6PD, and CS revealed subtype-dependent diagnostic performance, with greater discriminative capacity in triple-negative breast cancer, consistent with the elevated metabolic plasticity and energetic dependency characteristic of this subtype [29].

**Figure 11:**
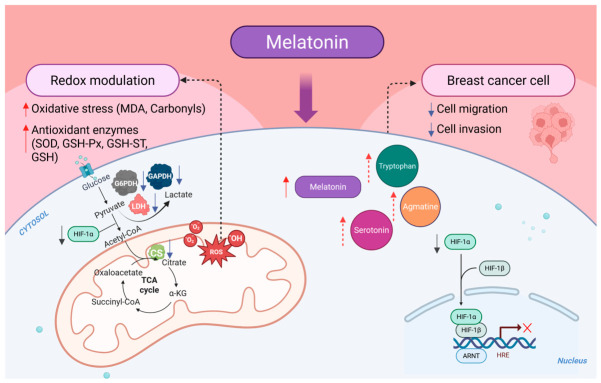
Schematic illustration of the main effects induced by melatonin in MCF-7 and MDA-MB-468 breast cancer cells. Blue arrows represent negative modulation, whereas red arrows indicate positive modulation of cellular and metabolic processes following treatment. Melatonin treatment increased the levels of tryptophan, serotonin, and agmatine, which show relevance in metabolic regulation and melatonin-related signaling pathways. Red dashed arrows indicate biochemical relationships based on established melatonergic pathways. Although these molecules were experimentally quantified in the present study, their enzymatic interconversion was not directly investigated. Created in BioRender. Cesário, R. (2026) https://BioRender.com/89h1fev (accessed on 11 June 2026).

Collectively, these findings support a model in which melatonin exerts coordinated intracellular effects by promoting mitochondrial metabolic repression, redox imbalance, and remodeling of biogenic amine profiles, ultimately leading to reduced cell viability, migration, and invasion (Figure 11). Importantly, the extent of these effects varies according to molecular subtype, reinforcing the influence of tumor metabolic context on melatonin-mediated antitumor activity. Further studies specifically addressing mitochondrial mechanisms will be essential to deepen the understanding of melatonin’s mode of action. This study has some limitations. First, the experimental design was restricted to in vitro models and therefore does not fully reproduce the complexity of the tumor microenvironment. Second, only luminal and triple-negative breast cancer cell lines were evaluated, whereas HER2-positive models were not included. Third, a non-tumoral mammary epithelial cell line was not evaluated in this study, preventing definitive conclusions regarding tumor selectivity. Finally, the experiments were conducted using a pharmacological concentration of melatonin selected from dose–response analyses to investigate antitumor mechanisms under experimental conditions. Although this approach is commonly used in mechanistic studies, additional investigations employing complementary preclinical models are necessary to further assess the translational relevance of these findings.

## 5. Conclusions

The present study demonstrates that melatonin exerts consistent antitumor effects in two breast cancer cell types (MCF-7 and MDA-MB-468) by impairing cell viability, migration, and invasion, while simultaneously modulating mitochondrial metabolism, redox balance, and intracellular biogenic amine profiles. Importantly, these effects were dependent on the molecular subtype, with more pronounced responses observed in MCF-7 (luminal) cells compared with MDA-MB-468 (triple-negative) cells. Together, these findings indicate that melatonin acts through coordinated metabolic and redox-related mechanisms that vary according to tumor molecular context, supporting further studies aimed at understanding its role in breast cancer metabolism and progression. The integration of functional, metabolic, mitochondrial, redox, and transcriptomic analyses provides a broader understanding of the mechanisms through which melatonin influences breast cancer biology across distinct molecular subtypes.

## Data Availability

The data presented in this study are available on request from the corresponding author. The data are not publicly available due to ongoing research.

## Supplementary Materials

The following supporting information can be downloaded at: https://www.mdpi.com/article/10.3390/cancers18132031/s1, Document S1: the uncropped WB images.

## Abbreviations

The following abbreviations are used in this manuscript:

ANOVA	Analysis of variance
AUC	Area under the curve
BSA	Bovine serum albumin
CAT	Catalase
CS	Citrate synthase
CT	Control
DAPI	4′,6-diamidino-2-phenylindole
DMEM	Dulbecco’s Modified Eagle Medium
DMSO	Dimethyl sulfoxide
ECL	Enhanced chemiluminescence
ER	Estrogen receptor
FBS	Fetal bovine serum
FPKM	Fragments per kilobase of transcript per million mapped reads
GAPDH	Glyceraldehyde-3-phosphate dehydrogenase
GSH	Reduced glutathione
GSH-Px	Glutathione peroxidase
GSH-ST	Glutathione S-transferase
G6PDH	Glucose-6-phosphate dehydrogenase
HER2	Human epidermal growth factor receptor 2
HIF-1α	Hypoxia-inducible factor 1-alpha
HPLC	High-performance liquid chromatography
IC_50_	Half maximal inhibitory concentration
IOD	Integrated optical density
LDH	Lactate dehydrogenase
MDA	Malondialdehyde
MEL	Melatonin-treated group
MTT	3-(4,5-Dimethylthiazol-2-yl)-2,5-diphenyltetrazolium bromide
PBS	Phosphate-buffered saline
PR	Progesterone receptor
ROC	Receiver operating characteristic
ROS	Reactive oxygen species
SEM	Standard error of the mean
SOD	Superoxide dismutase
TBS	Tris-buffered saline
TNBC	Triple-negative breast cancer
TNB	5-Thio-2-Nitrobenzoate
