# Supplementary material for: Antitumor Effects of Melatonin in Luminal and Triple-Negative Breast Cancer Cells: Metabolic Reprogramming, Redox Regulation, and Cellular Dynamics
Source: Cancers (Basel). 2026 Jun 23;18(13):2031. doi: 10.3390/cancers18132031 (PMC13359952; doi:10.3390/cancers18132031)

MCF-7

HIF1-α

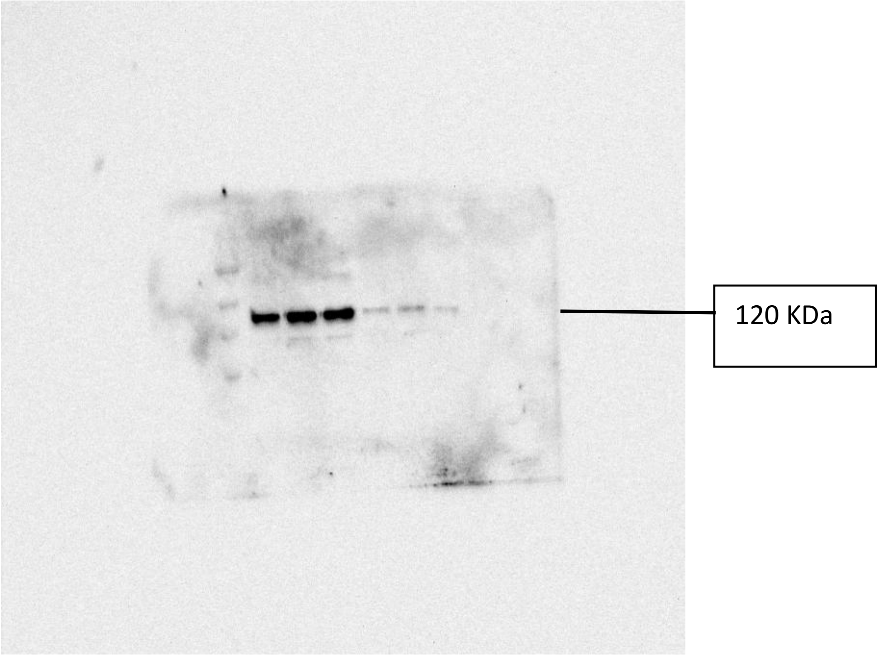

GAPDH

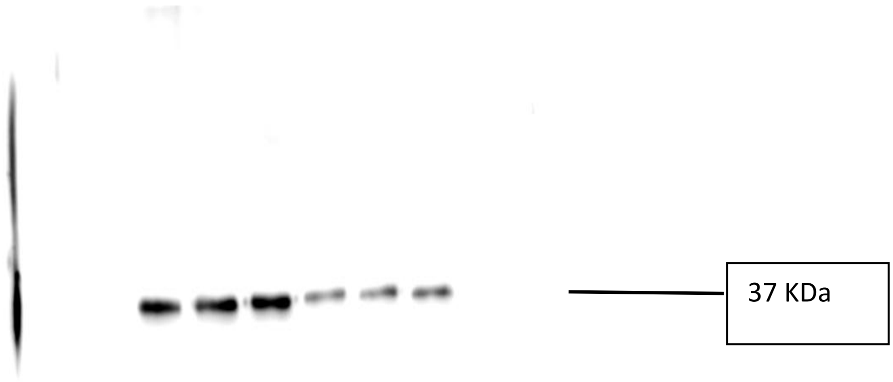

CS

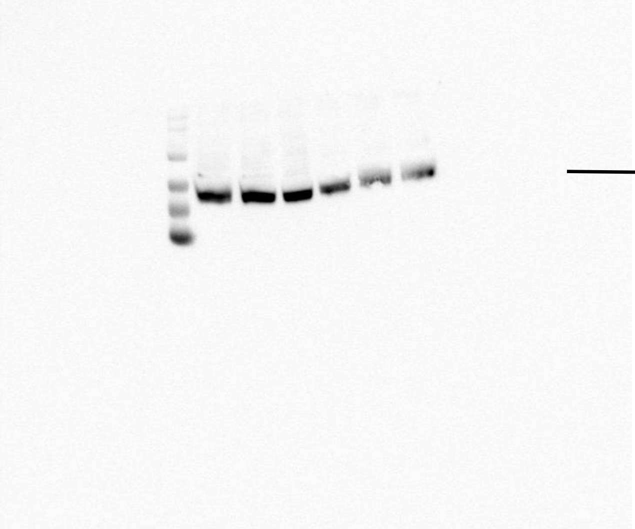

40 KDa

α-tubulin

55 KDa

MDA-MB-468

HIF1-α

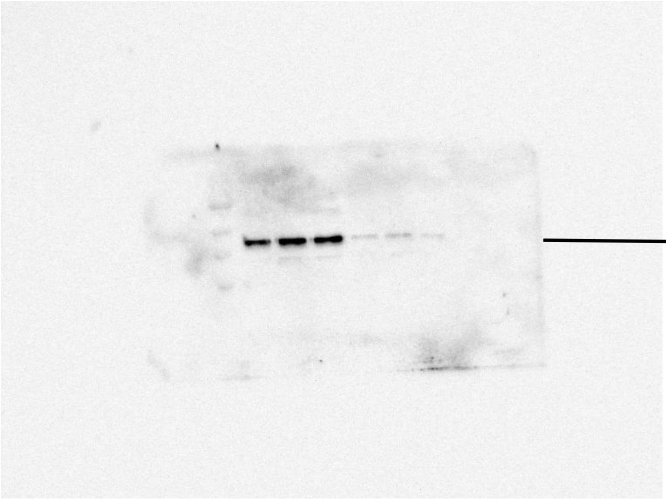

120 KDa

GAPDH

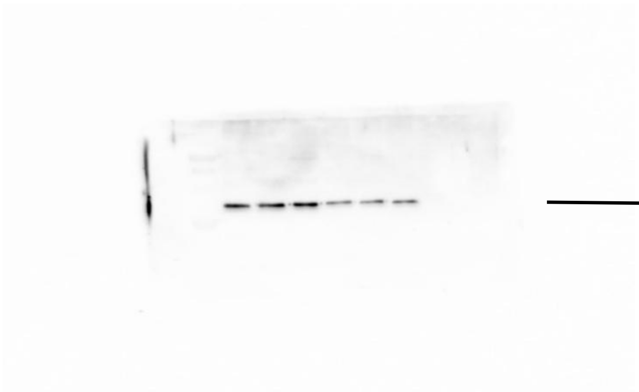

37 KDa

CS

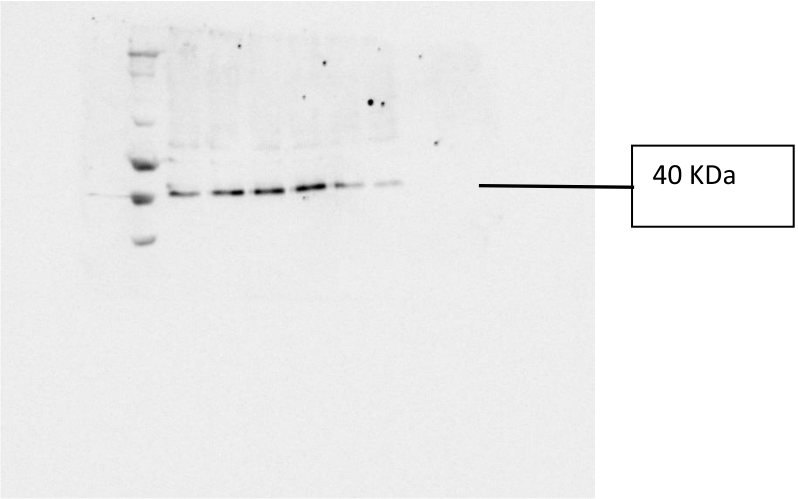

α-tubulin

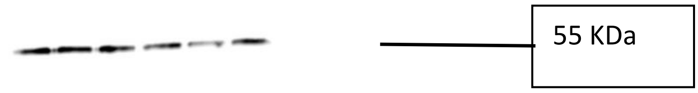

Supplement: Supplementary file 1 [file cancers-18-02031-s001.zip › cancers-4322971-supplementary.pdf]
